# Population genomics of the Japanese oak wilt pathogen reveals genetic structure mirroring its beetle vector and informs biosecurity risk assessment

**DOI:** 10.1186/s12864-026-13167-y

**Published:** 2026-07-17

**Authors:** John T. Dobbs, Rachel Koch-Bach, Christine Stone, Jo Anne Crouch

**Affiliations:** United States Department of Agriculture, Foreign Disease Weed Science Research Unit, Agricultural Research Service (USDA-ARS), Frederick, MD 21702 USA

**Keywords:** Japanese oak wilt, *Dryadomyces quercivorus*, Population genomics, *Platypus quercivorus*

## Abstract

**Background:**

Japanese oak wilt (JOW), caused by the fungus *Dryadomyces quercivorus*, has caused mass mortality of healthy oak trees in urban and landscape forests in Japan since the 2000s. The expanding geographic range of the pathogen, vectored by the ambrosia beetle *Platypus quercivorus*, threatens oak-dominated ecosystems and the timber industry globally. While variation in virulence among isolates has been described based on canker formation within Japanese oak species, there is limited understanding of the genetic variation of *D. quercivorus* that may underpin this virulence variation. Understanding the genetic variation represents a critical component to determining the capacity of this species to evolve novel virulences or overcome management strategies. In this study, improved hybrid assemblies of the JOW pathogen and closely related species including the Korean oak wilt pathogen (*D. quercus-mongolicae*) and the Mediterranean oak borer fungal symbiont (*D. montetyi*) were generated using Oxford Nanopore Technology long reads and Illumina short reads. To understand the genetic variation among JOW fungal populations, 32 isolates from two beetle lineages (A and B) across the Japanese archipelago were sequenced to assess population structure, genetic diversity, and evolutionary potential.

**Results:**

High-quality genomes were generated for *D. quercivorus*, *D. quercus-mongolicae*, and, for the first time, *D. montetyi*. DAPC, phylogenetic, and distance-based analyses, together with ADMIXTURE patterns at K = 2, revealed two biologically meaningful genetic groupings that broadly corresponded to beetle lineage. Isolates from beetle lineage B, primarily collected in a previous study on the eastern coast of Japan, clustered separately from most isolates associated with beetle lineage A, which were collected across other regions of Japan. Notably, this genetic structure did not correlate with previously reported virulence levels. Window-based Tajima’s D values were generally positive, a pattern consistent with weak bottlenecking and/or structured genetic variation rather than recent population expansion. Furthermore, evidence for sexual recombination was found. Both mating-type idiomorphs were present, and their overall frequency did not significantly deviate from a 1:1 ratio.

**Conclusions:**

This research shows that the major genetic structure observed in this ambrosia fungus broadly parallels that of its beetle vector lineages. This co-structuring, together with evidence consistent with sexual recombination, suggests that beetle-mediated introductions could transport multiple pathogen genotypes with the potential for recombination. This information is critical for biosecurity risk assessment and will be useful for tracking incursions of this pathogen outside of Japan and for developing effective management strategies, including the use of the genomic resources generated here for rapid diagnostics.

**Supplementary Information:**

The online version contains supplementary material available at https://doi.org/10.1186/s12864-026-13167-y.

## Background

Oaks (*Quercus* spp.) are foundational tree species, dominating ecosystems in North American forests, particularly in the eastern U.S. where they represent 11% of the total U.S. tree population and provide vital habitat and food sources for wildlife [1]. Oaks are one of the most economically important hardwoods, comprising 33% of all hardwood production in the U.S. in 2020 (https://www.americanhardwood.org/en/american-hardwood/american-white-oak). In Kentucky alone, white oak lumber generated around $61 million in 2018 [2]. However, North American forests are under constant threat from invasive foreign pathogens and pests. This local threat is part of a global crisis, with biological invasions recognized as a major driver of biodiversity decline and economic damage worldwide [3]. The economic toll of such invasions is immense, with recent estimates placing the cost in North America at over $26 billion per year since 2010 [4]. Within the forestry sector specifically, billions of dollars have been spent on responses to forest invaders, most of which were unsuccessful in eradicating the pathogen/pest [5].

The Japanese oak wilt (JOW) fungus, *Dryadomyces quercivorus* (Kubono & Shin. Ito) M. Procter & Z.W. de Beer (Basionym: *Raffaelea quercivora* Kubono & Shin. lto) was first discovered in Japan in the 1980s [6, 7] and has been confirmed to cause disease on 12 tree species in the Fagaceae, mainly Japanese oak species, *Quercus mongolica* subsp. *crispula* (Blume) Menitsky (Basionym: *Q. crispula* Blume) and *Q. serrata* Murray [7–9]. Affected trees exhibit wilting and discoloration of foliage, as well as stained and non-conductive sapwood where the pathogen is consistently isolated [8, 9]. JOW outbreaks have changed ecosystem dynamics and caused extensive loss of valuable forest resources in Japan [10, 11]. Similarly, the Korean oak wilt (KOW) pathogen, *D. quercus-mongolicae* (K. H [12]). M. Procter & Z.W. de Beer (Basionym: *R. quercus-mongolicae* K. H [12]). has caused severe damage and death to healthy oak trees in urban and landscape forests in Korea since 2004 [13]. Both pathogens are spread by ambrosia beetle vectors, *Platypus quercivorus* Murayama and *P. koryoensis* Wood & Bright, respectively. The beetle vectors have both short- and long-distance (up to 27 km) dispersal phases, with most beetles undergoing short dispersals to nearby host trees where they mate and reproduce, surviving on the wood and an associated yeast (*Ambrosiozyma kashinagacola* [Endoh, M. Suzuki, Benno & Futai] Kurtzman & Robnett) farmed within the gallery for food [14, 15]. However, they may be spread through several pathways associated with global trade. The importation of live plants for planting is recognized as a major pathway for the establishment of new forest pests and pathogens in the U.S. [16], though risks are also associated with untreated wood packing material, such as crates or pallets, and firewood [17]. Given the scale of the live plant trade, there is real concern that the introduction of either of these foreign pathogens to North America could have severe consequences for U.S. eastern oak forests because the eastern U.S. and eastern Asia have similar temperate biomes conducive to the survival of the beetle vector and the pathogen.

A critical component of risk assessment is understanding the potential host range and evolutionary potential of an invasive pathogen. Only one North American oak species (*Q. rubra*) has been tested for susceptibility to the JOW pathogen [18], and no North American oak species have been tested for susceptibility to the KOW pathogen. This knowledge gap is especially concerning given the demonstrated ability of ambrosia beetle-vectored pathogens to jump to novel hosts of high conservation value; for example, the laurel wilt pathogen was discovered infecting the federally threatened pondberry (*Lindera melissifolia* Blume) [19]. This risk is further complicated by observed variation in pathogen virulence. A previous study conducted virulence assays of 40 JOW isolates on Japanese oak species (*Quercus serrata* and *Q. crispula*) and found significant variation in virulence based on canker lengths [20]. This variation suggests that similar to other ascomycete fungi like *Fusarium* spp., populations of *D. quercivorus* may contain both highly virulent and less-aggressive strains [21, 22]. Furthermore, evidence of host-specific interactions exists, as pathogenicity tests on various *Fagaceae* species in Japan have revealed differing levels of susceptibility [23]. However, the genomic basis for this virulence variation remains unknown. Understanding the genomic differences between the highly virulent and less-aggressive *D. quercivorus* could provide key insights into the evolution of virulence and aggressiveness in this system.

Due to the unique relationship among these fungi and their beetle vectors, the spread of these pathogens is highly dependent on the beetles’ ability to enter a suitable host tree. The female beetles cultivate a symbiotic yeast (*A. kashinagacola*) within the gallery walls for the larvae to consume; additionally, the female beetles also cultivate the JOW pathogen, but it is not consumed as a food source [24, 25]. This is unlike other ambrosia beetles that cultivate their fungal symbionts for food, such as the redbay ambrosia beetle (*Xyleborus glabratus* Eichhoff) with *Harringtonia lauricola* (T.C. Harr., Fraedrich & Aghayeva) Z.W. de Beer & M. Procter [26] and the Mediterranean oak borer (MOB; *Xyleborus monographus* Fabricius) with its nutritional symbiont *Dryadomyces montetyi* (M. Morelet) M. Procter & Z.W. de Beer (Basionym: *Raffaelea montetyi* M. Morelet) [27, 28]. Recent genomic analysis of *P. quercivorus* identified two genetic lineages in Japan, with Group A distributed nationwide and Group B reported as restricted to south and southwest Japan [25, 26]. However, Group B was found in a previous study localized to the eastern coast of Japan [20]. While the population structure of the vector has been described, a critical unanswered question is whether the pathogenic fungus, *D. quercivorus*, also follows this pattern of co-structuring with its vector. The JOW pathogen produces compounds that cause xylem to lose its water-conducting function, which leads to xylem dysfunction, wilting, and death of susceptible trees [8, 9, 23, 29]. Comparative genetics and genomics may provide a means to identify the mechanisms for establishment within the host used by the highly virulent strains of these oak wilt fungi that ultimately causes xylem dysfunction [30].

Limited research is available on the genetic and genomic comparisons of putative populations of the JOW pathogen. Conserved nuclear ribosomal DNA sequence data has been used to identify the phylogenetic relationship between JOW isolates and closely related species [31]. Diverse JOW pathogen genotypes have been observed among the beetle vector and their galleries within the tree based on simple sequence repeat (SSR) markers [32]. However, these markers offer limited resolution. Using whole genome sequencing to identify genome-wide single nucleotide polymorphisms (SNPs) enables a more comprehensive assessment of genetic variation in populations. Compared to SSRs, SNP data can improve the resolution of datasets when using small sample sizes [33, 34]. SNPs also provide a less biased estimate of population-level diversity and greater power to identify genetic groups [35, 36]. Additionally, including comparative genomics among genetic variants can be used to identify genes that are associated with pathogenicity, virulence, and host specificity [37].

Since the JOW and KOW pathogens are not known to be established in North America, understanding the genetic and genomic variation of these pathogens is a critical step toward informing biosecurity risk assessments and preparing for a potential invasion. Global assessments have highlighted that data on alien species is often scattered and incomplete [3]; therefore, generating detailed, high-resolution genomic data for high-risk pathogens like *D. quercivorus* is essential to fill these critical knowledge gaps. In this research, our objectives were to (1) improve genomic resources for the JOW, KOW, and MOB pathogens for use in population and comparative genomics; (2) determine if the population structure of the JOW pathogens is driven by geography or beetle vector lineage; (3) identify if these populations correlate with virulence; and (4) identify putative pathogenicity/virulence genes among these populations that could explain variation in virulence among JOW pathogen isolates.

## Methods

### Fungal isolates

*Dryadomyces quercivorus* isolates (*n* = 33) used in this work were obtained from the Centraalbureau voor Schimmelcultures (CBS) collection (Westerdijk Fungal Biodiversity Institute; Utrecht, The Netherlands), the National Agriculture and Food Research Organization (NARO) Research Center of Genetic Resources, and colleagues at the Tohoku Research Center, Forestry and Forest Products Research Institute (Iwate, Japan) (Supplemental Table 1). *Dryadomyces quercus-mongolicae* (KACC44403, KACC44404, and KACC44405) isolates were obtained from the Korean Agricultural Culture Collection (KACC), while a culture of another relative within the *Dryadomyces* genus, *Dryadomyces montetyi* (CBS463.94), was obtained from CBS (Supplemental Table 1).

### Whole genome sequencing

Isolates were cultured for 7 days at room temperature under 12 h fluorescent lighting in 30 ml of 20% clarified V8 broth prepared similar to Miller [38] without the addition of agar and diluted to 20% using sterile water. Total DNA was extracted using a cetyltrimethylammonium bromide (CTAB) extraction protocol adapted from Dobbs et al. [22]. Briefly, mycelial tissue was separated from liquid broth using a Buchner funnel under vacuum on Whatman no. 1 filter paper (Cytiva, Wilmington, DE). Tissue was collected into 2 ml microcentrifuge tubes and frozen at -80 °C for 24 h prior to lyophilization for 3 h. Dried tissue was ground to a fine powder using liquid nitrogen in a mortar and pestle. One ml of prewarmed (60 °C) CTAB extraction buffer (OPS Diagnostics LLC, Lebanon, NJ) was added to the sample and homogenized in a TissueLyser III (Qiagen, Hilden, Germany) for 1 min at 30 Hz. Tubes were centrifuged at 15,000 rcf for 5 min to pellet cellular debris and the supernatant was transferred to another tube (ca. 800 µl). RNase A (10 mg/ml; 20 uL; Thermo Fisher Scientific, Waltham, MA) and proteinase K (20 mg/ml; 10 uL; Qiagen, Hilden, Germany) were added to each tube and incubated at 55 °C for 1 h with agitation at 450 rpm in a Thermomixer R (Eppendorf, Hamburg, Germany). One volume of chloroform: isoamyl alcohol (24:1) was added to each tube. Tubes were mixed by inversion for 5 min at 30 rpm orbital in a HulaMixer (Thermo Fisher Scientific, Waltham, MA) and then centrifuged at 14,000 rcf for 2 min. Supernatant (600 µl) was transferred to new 2 ml tubes. DNA was precipitated using one volume (600 µl) of room-temperature isopropanol and inverted gently 10 times to mix and centrifuged at 16,000 rcf for 2 min at 20 °C. Supernatant was removed and the pellet was washed twice using 1 ml of ice cold 70% ethanol and centrifuged at 19,000 rcf for 5 min at 4 °C. The DNA pellet was dried for 3 min using a Vacufuge Plus Concentrator (Eppendorf, Hamburg, Germany) to remove any excess ethanol. DNA was resuspended in 60 µl molecular biology grade water. DNA quality and integrity was assessed using a NanoDrop Lite Plus (Thermo Fisher Scientific Inc., Waltham, MA) and a 1% agarose gel electrophoresis. DNA was quantified using a Qubit 4 Fluorometer (Invitrogen, Waltham, MA). Extracted DNA was sent to Novogene (Davis, CA, USA) for TruSeq Illumina library preparation and sequencing with paired-end reads of 2 × 151 bp on an Illumina NovaSeq platform. Paired-end reads were trimmed using fastp [39] for use in population genomics and comparative genomics analyses.

### Whole genome assembly

Trimmed paired-end reads for each isolate were used for *de novo* genome assemblies using SPAdes v4.2.0 [40] for comparative genomics analysis. Additionally, to improve genomic resources of these important plant pathogens, high-quality genomes were constructed using hybrid assemblies of three representative isolates of the JOW pathogen and closely related *Dryadomyces* spp. (*D. quercus-mongolicae* and *D. montetyi*) using the Illumina 150 PE reads described above and Oxford Nanopore Technologies (ONT) long-read sequencing (Supplemental Table 1). For ONT genome sequencing, DNA was extracted as described above and was additionally processed using a size selection protocol described in Jones et al. [41] using size selection solution (4% PVP 360,000, 1.2 M KCl, and 20mM Tris-HCl pH 8 in nuclease free water) to preferentially sequence reads > 10 kb. Libraries were generated using the ONT Native barcoding kit v14 (SQK-NBD114-96) following the manufacturer’s protocols with minor edits. Sequencing was conducted on a PromethION P2 Solo using R10.4.1 PromethION flow cells on a MacStudio Pro for approximately 72 h. Raw reads were basecalled using dorado v1.3.0 using the model dna_r10.4.1_e8.2_400bps_sup@v5.0.0 (https://github.com/nanoporetech/dorado). Read quality and length trimming (> 1 kb) was conducted using the program fastplong [39]. Genomes were assembled using the MaSuRCA (Maryland Super-Read Celera Assembler) v4.1.4 pipeline [42]. Mitochondrial reads were removed using GetOrganelle [43] using the fungus_mt database from the assembly graph with default parameters. Reads that mapped to the mitochondrial contigs generated from GetOrganelle were removed and MaSuRCA was rerun using those filtered reads. The genome assembly was polished with the nanopore reads using three iterations of Racon v1.5.0 [44] using minimap2 (-ax map-ont) [45] to map reads to the assembly prior to each Racon iteration (-m 8 -x -6 -g -8 -w 500 -t 14). This was followed by polishing with Medaka v2.2.0 (https://github.com/nanoporetech/medaka) using medaka_consensus with default parameters, and a final polishing with POLCA [46] using the trimmed and filtered Illumina reads. BUSCO v6.0.0 [47] was used to search against the fungi_odb12 and ascomycota_odb12 databases and QUAST [48] was used to assess the completeness and quality of assembled genomes.

National Center for Biotechnology Information (NCBI) reference genomes of the JOW (Accession: GCA_002778125.1) and KOW (Accession: GCA_002215975.1) pathogens were used for comparison with the genomes generated in this study. For the JOW holotype isolate used in this study, JOW1.CBS122982, previously published Pacific Biosystems (PacBio) Single Molecule, Real-Time (SMRT) genomic reads were collected from GenBank (Accession: SRR5865558) [49]. The PacBio SMRT reads were concatenated with the ONT reads and used as the long read-input for the MaSuRCA pipeline described above. The resulting long-read assembly was then polished using POLCA with the Illumina reads. The completeness and contiguity of the final assembly were assessed using BUSCO and QUAST using methods described above.

### Genome annotation

Genomes were annotated using BRAKER v3.0.8 [50]. Genomes were soft-masked using RepeatModeler v2.0.7 [51] and RepeatMasker v4.2.2 [52] using the Dfam database v3.8 [53]. These soft-masked assemblies were used as input for BRAKER along with fungal proteins from OrthoDB v12 as extrinsic evidence [54]. Previously published RNA-seq Illumina HiSeq reads of total RNA in culture (Accession: SRR5865579) from the JOW isolate [49] and the KOW holotype isolate used in this study, KOW3.KACC44405 (Accessions: SRR32758122, SRR32757902, and SRR32757901) [55] were used as evidence in the genome annotation for their respective species. The output GTF file was used for identification of gene positions for population structure analysis. EggNog-mapper v2.1.13 [56], Phobius v1.01 [57], Signalp v6.0 [58], TargetP v2.0 [59], TMHMM v2.0 [60], DeepLoc v2.1 [61], EffectorP v3.0 [62], and PHIbase v4.18 [63] were used to identify putative gene families including protein family domains, carbohydrate active enzymes (CAZymes), secreted proteins and effectors, and pathogen-host interaction genes (PHI-base homologs). Proteins were considered secreted if they contained a signal peptide, lacked transmembrane domains, and were predicted to have an extracellular localization (Supplemental Table 1).

### Comparative genomics

Annotated genomes were assessed for similarity of virulence genes among populations and highly virulent isolates. OrthoFinder v3.1.2 [64] was used to identify orthologous proteins from two datasets analyzed separately: (1) all predicted proteins, and (2) a curated subset of predicted secreted effectors, including carbohydrate-active enzymes (CAZymes) and pathogen-host interaction genes (PHI-base homologs). The relationship between orthogroup abundance and isolate traits was visualized using principal coordinates analyses (PCoA) using Bray–Curtis dissimilarities. We then tested for significant associations using permutational multivariate analysis of variance (PERMANOVA) between orthogroup composition and putative populations, beetle lineage, and virulence rating (based on canker length in [20]) using Bray–Curtis distance matrices implemented in RStudio using the ‘vegan’ package [65]. Virulence was assessed using previously published canker-length data for overlapping isolates from [20, 66]. For analyses requiring a categorical virulence variable, isolates were classified according to whether their mean canker length was reported as significantly different from the control in the original study. PERMANOVA tests involving virulence were restricted to isolates for which published canker-length data were available.

To assess the potential for sexual reproduction and provide biological context for the population genomics analyses, mating type idiomorphs (*MAT1-1-3* and *MAT1-2-1*) were identified in each genome assembly. Mating type-loci were identified bioinformatically from the genome assemblies. Specifically, the conserved mating-type flanking proteins APN2 (P38207) and SLA2 (P33338) from *Saccharomyces cerevisiae* were used as queries in a tBLASTn search [67] to locate the putative mating type locus in each isolate. To further confirm locus identity, previously published degenerate primers for MAT loci [68] identified in *D. quercus-mongolicae* were mapped to each genome assembly using Primer-BLAST [69] within Geneious Prime (Biomatters Ltd.). Candidate loci were then verified by tBLASTx searches against the NCBI nr database to confirm the presence of conserved domains characteristic of ascomycete mating type genes including the alpha-box domain for *MAT1-1* and the high mobility group (HMG)-box domain for *MAT1-2* [68]. Each isolate was classified as containing either the *MAT1-1-3* or *MAT1-2-1* idiomorph. The ratio of isolates containing each idiomorph was tested for deviation from a 1:1 ratio with an exact binomial test in RStudio due to the small sample size.

### Population genomic alignment and variant calling

Our hybrid assembly of the holotype isolate, JOW1.CBS122982, was selected as the reference genome for all subsequent analyses because it was the most complete and contiguous assembly based on BUSCO and QUAST results. Trimmed Illumina reads from the additional JOW isolates were aligned to the reference genome assembly using BWA v2.2.1 [70] with the option bwa mem. The resulting SAM files were converted to BAM files and indexed and sorted using SAMtools v1.17 [71]. Filtering and variant calling was conducted using the Broad Institute’s Genome Analysis Toolkit (GATK) v4.4.0.0 best practices pipeline [72]. Generated BAM files were quality checked with the option ValidateSamFile. Duplicate reads were marked and removed using Picard v3.0.0 [73] with the option MarkDuplicatesSpark. Read depth was calculated using SAMtools depth. Variants were called using GATK HaplotypeCaller and combined using CombineGVCFs before genotyping using GenotypeGVCFs. Single nucleotide polymorphisms (SNPs) and indels were extracted and filtered using GATK SelectVariants and VariantFiltration. SNPs and indels were excluded separately, and only variants that passed all quality filters were retained for downstream analysis using the SelectVariants “--exclude-filtered” flag. A two-pass base quality score recalibration (BQSR) was conducted for each isolate. First, BaseRecalibrator was run using the known SNP and indel sites generated from the combined VCFs. Second, ApplyBQSR was used to apply the recalibration, and then BaseRecalibrator and AnalyzeCovariates were run again to evaluate the results. Variants were called again and SNPs and indels were extracted and filtered as described above. SNP locations within the genome and predicted effects on predicted genes were assessed using snpEff v5.2f [74] using the BRAKER GTF file as input. Plink2 [75] was used to filter the resulting VCF file for only biallelic variants using the “--max-alleles 2” and “--make-bed” parameters.

### Phylogenetic and population genetic analyses

A maximum-likelihood (ML) phylogeny was generated using IQ-Tree v2.4.0 [76] to assess the phylogenetic relationships among JOW isolates. The trimmed paired-end Illumina reads were mapped to the reference genome using the program snippy [77]. An alignment was generated based on the core SNPs using the “snippy-core” command. A core site in the program is defined as a genomic position that is present in all the samples that is either monomorphic or polymorphic while ignoring insertions and deletion variant types. The best-fit nucleotide substitution model with correction for ascertainment bias was determined using ModelFinder [78] as implemented in IQ-Tree using the “-m MFP + ASC” flag. Tree branch support was assessed via 1000 ultrafast bootstrap replicates [79], and the “-bnni” flag was used to perform a hill-climbing nearest neighbor interchange search to reduce the risk of overestimating branch supports using the “-bnni” flag.

### Population structure analysis and spatial analysis

To define a priori populations for downstream analyses, isolates were grouped by beetle lineage (Group A or Group B) as the primary population level and by seven geographical regions (north, west coast, central, east coast, Miyake Island, southwest, and south) as a secondary, subpopulation level. To test for evidence of recombination, which can influence population structure analyses, the pairwise homoplasy index (PHI) test [80] was performed in SplitsTree v6.7.2 [81]. This test used a distance matrix generated with the P distance method [82]. SplitsTree phylogenetic network was generated to investigate the signal under recombination. Tajima’s D was calculated in R using the vcfR package using only filtered biallelic SNPs. Analyses were performed separately for each region and beetle lineage. Tajima’s D was calculated in non-overlapping 10 kb windows with a 10 kb step size across each contig. Windows with fewer than 2 segregating sites were excluded from summary statistics. For each region or beetle lineage, the reported Tajima’s D value represents the mean Tajima’s D across all valid windows. The overall Tajima’s D was calculated as the arithmetic mean of the beetle lineage populations-specific medians and was not estimated from pooled samples. The overall Tajima’s D was calculated from population-level summaries rather than from a pooled analysis of all individuals, because pooling structured populations can bias Tajima’s D by increasing the frequency of intermediate-frequency variants. An Analysis of Molecular Variance (AMOVA) was performed using the R package ‘poppr’ [83], which was also used to generate a minimum spanning network (MSN) to evaluate sample-level genetic connectivity based on bitwise distance. Clone correction was evaluated using multilocus genotype (MLG) assignment in poppr to determine whether repeated genotypes were present in the dataset.

Four clustering analyses were used to examine JOW population structure. Data were subjected to Principal Component Analysis (PCA) and Discriminant Analysis of Principal Components (DAPC) within the R package Adegenet v2.1.3 [84, 85]. PCA and DAPC analyses were conducted on the biallelic filtered VCF file and analyzed separately for beetle lineage and geographical regions. Additionally, multiple factor analysis (MFA) was conducted to simultaneously analyze the effect of beetle lineage and geographical region using factoextra v2.0.0 [86] in RStudio. For MFA, SNP-derived genetic coordinates were analyzed together with categorical metadata for beetle lineage and geographic region to visualize whether clustering patterns were jointly associated with both explanatory variables. In parallel, ADMIXTURE v1.3.0 [87] was performed with cross-validation (CV) to estimate the optimal number of populations (K) ranging from 1 to 7 to estimate the optimal number of populations. At each value of K, ADMIXTURE was run ten times using the filtered VCF with unique starting random seed numbers. Results were plotted using the ‘ggplot2’ package v3.5.0 in RStudio. The optimal number of K was determined using the lowest average CV value. To test for spatially autocorrelated genetic distances that may be impacting ADMIXTURE analyses, a linear regression, Mantel test, and multiple regression on distance matrices (MRM) were conducted in RStudio using base R, ‘vegan’, and ‘ecodist’ v2.1.3 [88] R packages, respectively. Genetic distance matrix for these analyses was constructed using ‘poppr::bitwise.dist.’ Geographic distances among isolates were estimated from approximate sampling coordinates (latitude and longitude) obtained in Google Earth based on the reported collection locality for each isolate (Supplemental Table 1). Pairwise great-circle distances were then calculated in R using the Haversine method. Beetle lineage distances were calculated as a binary choice of 0 indicating the same lineage and 1 indicating a different lineage. Mantel test was conducted for genetic distances compared to geographic distance and beetle lineage separately as well as to test if beetle lineage and geographic region are strongly correlated with each other. Significance for Mantel and MRM tests was assessed using 9,999 permutations.

## Results

### High-quality genomes for Dryadomyces pathogens

Our hybrid assembly of the JOW pathogen holotype (JOW1.CBS122982; GenBank Accession: JCAECF010000000), which integrated ONT, PacBio SMRT, and Illumina reads, yielded a highly contiguous 26 Mb genome assembled into nine contigs. This assembly showed a BUSCO completeness of 99.2% (fungi_odb12) and 98.1% (ascomycota_odb12) (Table 1). The hybrid assembly of the JOW pathogen’s paratype isolate (MAFF410919; GenBank Accession: JCAECD010000000) resulted in a similarly high-quality genome with 10 contigs, a genome size of 26 Mb, and BUSCO completeness of 98.9% (fungi_odb12) and 98.0% (ascomycota_odb12) (Supplemental Table 1). The additional isolate (JOW11.RA1541; GenBank Accession: JCAECE010000000) was assembled into 22 contigs with a genome size of 26 Mb, and BUSCO completeness of 99.0% (fungi_odb12) and 97.9% (ascomycota_odb12) (Supplemental Table 1).

**Table 1:** Comparison of NCBI reference genomes of the *Dryadomyces quercivorus* type specimen (JOW1.CBS122982), *Dryadomyces quercus-mongolicae* type specimen (KOW3.KACC44405), and the genomes generated in this study. Additionally, assembly statistics of the *Dryadomyces montetyi* type specimen (CBS463.94) with no reference for comparison

	JOW1.CBS122982 NCBI Reference	JOW1.CBS122982 This Study	KOW3.KACC44405 NCBI reference	KOW3.KACC44405 This study	CBS463.94 This study
Genome size (bp)	26,114,139	26,012,413	27,005,990	27,169,684	26,631,764
Number of contigs	23	9	43	11	10
Number of contigs > = 50 kb	10	7	17	8	8
N50	3,691,412	3,837,608	2,234,633	4,661,137	4,332,708
Coverage	139x	219x	5,965x	143x	432x
GC content	57.02%	56.97%	54.25%	54.26%	55.30%
Genome completeness – fungi_odb12	C:99.2% [S:98.8%, D:0.4%], F:0.1%, M:0.7%, n:1122, E:29.6%	C:99.2% [S:99.2%, D:0.0%], F:0.1%, M:0.7%, n:1122, E:29.6%	C:99.1% [S:99.1%, D:0.0%], F:0.1%, M:0.8%, n:1122, E:28.7%	C:99.1% [S:99.1%, D:0.0%], F:0.1%, M:0.8%, n:1122, E:28.8%	C:99.3% [S:99.3%, D:0.0%], F:0.3%, M:0.4%, n:1122, E:29.9%
Genome completeness – ascomycota_odb12	C:97.9% [S:97.7%, D:0.3%], F:0.4%, M:1.6%, n:2826, E:27.3%	C:98.1% [S:97.9%, D:0.1%], F:0.4%, M:1.6%, n:2826, E:27.2%	C:97.7% [S:97.5%, D:0.1%], F:0.5%, M:1.8%, n:2826, E:26.6%	C:97.7% [S:97.6%, D:0.1%], F:0.5%, M:1.8%, n:2826, E:26.5%	C:98.3% [S:98.2%, D:0.1%], F:0.3%, M:1.4%, n:2826, E:26.6%

The hybrid assembly of the *D. quercus-mongolicae* holotype (KACC44405; GenBank Accession: JCAECA010000000) resulted in a 27.1 Mb genome assembled into 11 contigs and BUSCO completeness of 99.1% (fungi_odb12) and 97.7% (ascomycota_odb12) (Table 1). While the current NCBI reference genome (which only used Illumina reads) is similarly complete, our new assembly significantly improved contiguity by incorporating ONT long reads. The assemblies for the *D. quercus-mongolicae* paratype isolates yielded 9 and 10 contigs with a genome size of 27 Mb and BUSCO completeness of 99.3% (fungi_odb12) and 97.7 and 97.5% (ascomycota_odb12), for isolate KACC44403 (GenBank Accession: JCAECC010000000) and KACC44404 (GenBank Accession: JCAECB010000000), respectively (Supplemental Table 1). The assembly of the holotype of *D. montetyi* (CBS463.94; GenBank Accession: JCAECG010000000) resulted in a 26.6 Mb genome in 11 contigs and BUSCO completeness of 99.3% (fungi_odb12) and 98.3% (ascomycota_odb12) (Table 1). There is currently no available reference genome on NCBI for comparison, making this the first publicly available genome for this species.

Illumina sequencing of the 33 *D. quercivorus* isolates generated an average of 19 million reads per isolate, totaling approximately 3 Gb of data (Supplemental Table 1). *De novo* assembly of these reads using SPAdes produced high-quality draft genomes with an average of 72 contigs and an average BUSCO completeness of 98.9 and 97.9% against the fungi_odb12 and ascomycota_odb12 databases, respectively (Supplemental Table 1).

### Population genetics and mating type distribution

**Fig. 1:**
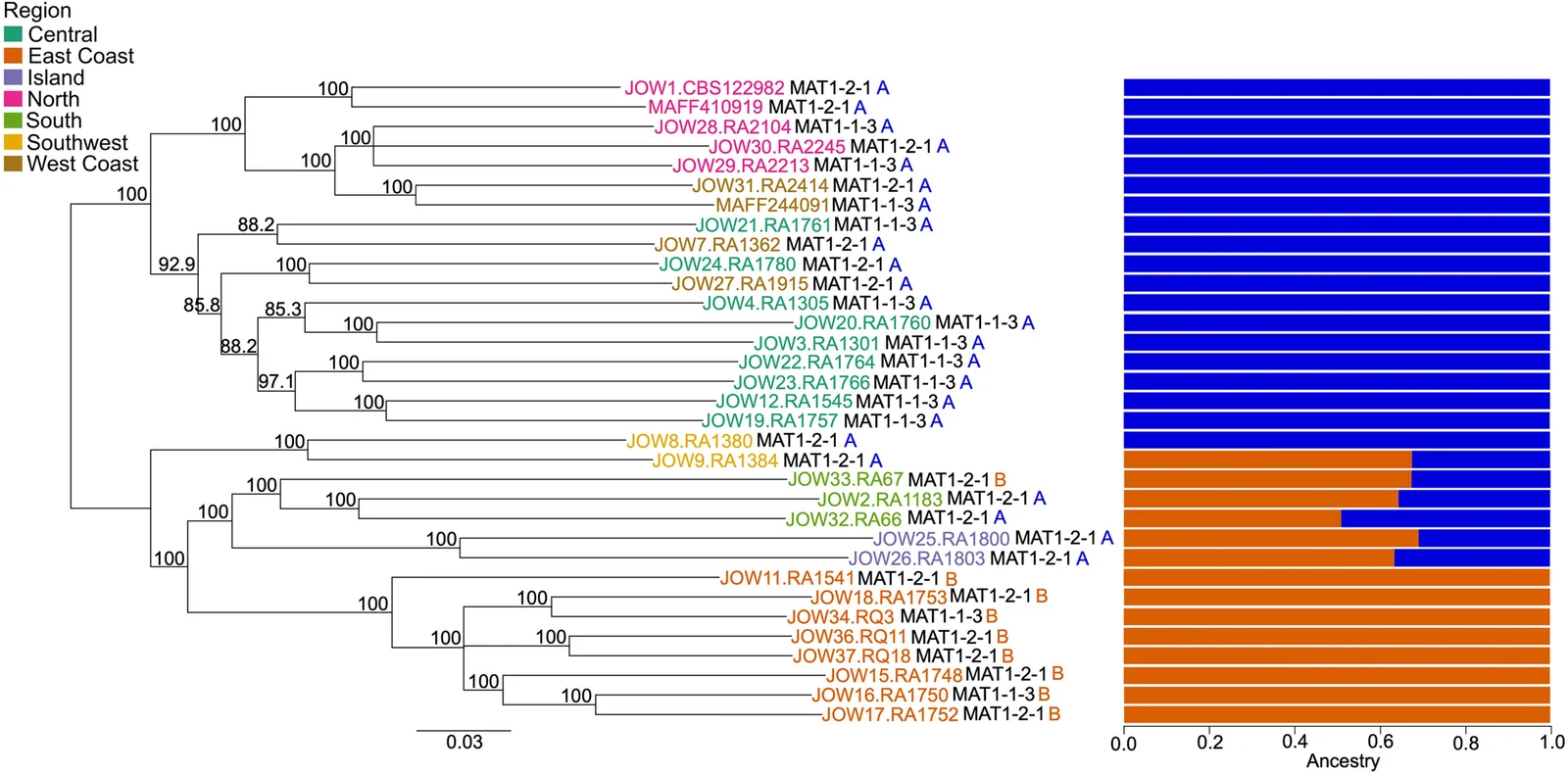
Population structure of *Dryadomyces quercivorus* broadly corresponds to its beetle vector lineage. Maximum likelihood phylogeny of 33 isolates based on 292,996 single nucleotide polymorphisms is annotated with isolate metadata including mating type idiomorph (*MAT1-1* or *MAT1-2*) and beetle vector lineage (lineage A or lineage B). The ADMIXTURE plot displays population assignments at K = 2, showing two primary clusters (blue and orange). Bootstrap support values > 70% are shown at the nodes. The tree is rooted with isolates JOW8.RA1380 and JOW9.RA1384 for visualization

Among the 32 JOW isolates (excluding the reference), aligning reads to our reference genome initially identified 3,683,607 unfiltered variants. After stringent filtering, 217,240 SNPs remained for downstream analysis. Among these SNPs, 156,766 were found in SplitsTree across all isolates based on their regional differences (Supplemental Fig. 1). We observed differences in the number of unique SNPs between groups; for example, isolates from beetle lineage B had fewer unique SNPs (26,832) than those from lineage A (103,111), consistent with greater within-group sequence variation in the A lineage. Phylogenetic analysis of the core SNP alignment (292,996) generated by snippy revealed two main clades that corresponded with the beetle vector lineage. One clade consisted entirely of isolates collected from beetle lineage B in the “east coast” region, while another clade comprised isolates entirely from beetle lineage A collected throughout Japan (Fig. 1). The remaining clades consisted of isolates primarily collected from beetle lineage A with the exception of one isolate collected from beetle lineage B in the “south” region of Japan (Fig. 1).

The mating-type flanking genes *APN2* and *SLA2* were identified in all *de novo* assembled and reference genomes tested in this study. Each JOW isolate was found to possess either the *MAT1-1-3* or the *MAT1-2-1* idiomorph, but never both. The *MAT1-2-1* idiomorph was identified in 60.6% of the JOW isolates (*n* = 20 out of 33 isolates) and the *MAT1-1-3* idiomorph was identified in 39.4% of isolates (*n* = 13 out of 33 isolates) (Table 2). The KOW and *D. montetyi* isolates were also predicted to have the *MAT1-2-1* locus (Supplemental Table 1). Beetle lineage A had 13 *MAT1-2-1* and 11 *MAT1-1-3* isolates. Beetle lineage B had 7 *MAT1-2-1* and 2 *MAT1-1-3* isolates. An exact binomial test showed that these ratios did not indicate a significant deviation from the expected 1:1 ratio in the overall population or within most subgroups, with one exception: the “central” region showed a significant deviation from 1:1 (*p* = 0.03906), consisting almost entirely of isolates with the *MAT1-1-3* locus (Table 2).

**Table 2:** Summary statistics for genome-wide biallelic single nucleotide polymorphisms (SNPs) in *Dryadomyces quercivorus* grouped by beetle lineage and geographic region. Total SNPs represent segregating SNPs within each grouping, while unique SNPs represent SNPs observed only within that grouping. Tajima’s D values summarize non-overlapping 10 kb windows containing at least two segregating sites

Group	Number of Isolates	Total SNPs	Unique SNPs	Mean Tajima’s D	MAT1-1-3	MAT1-2-1
Overall	33	217,240	N/A	0.4089	13	20
Beetle lineage A	24	190,408	103,111	0.0195	11	13
Beetle lineage B	9	114,129	26,832	0.6830	2	7
North	5	71,507	6,319	1.0664	2	3
West coast	4	81,151	5,901	1.0655	1	3
Central	9	118,851	20,505	0.6775	8	1
East coast	8	98,389	20,757	0.9402	2	6
Miyake island	2	70,719	13,685	2.2015	0	2
Southwest	2	33,983	4,535	1.3361	0	2
South	3	87,123	17,022	2.1117	0	3

### Population structure

**Fig. 2:**
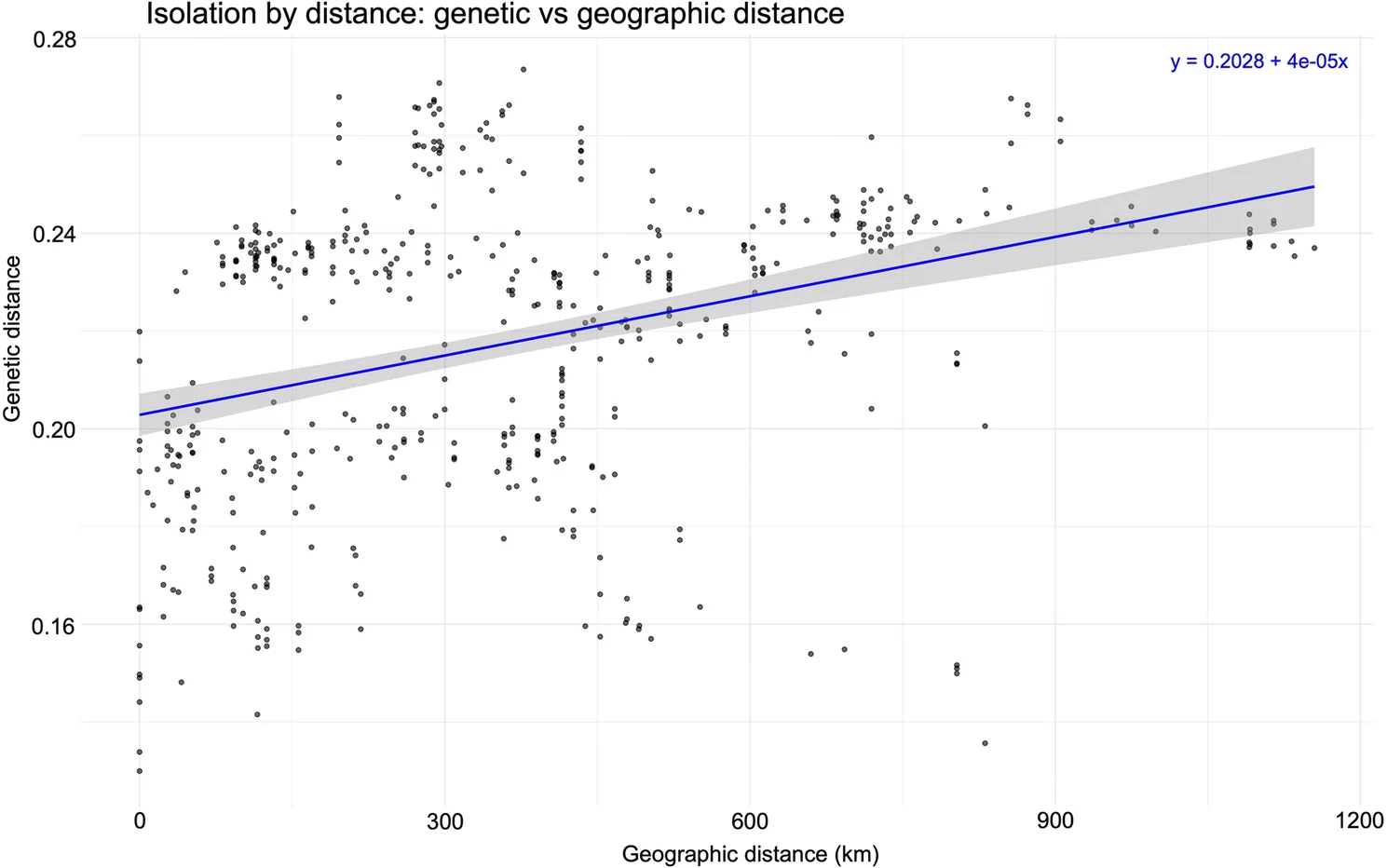
Linear regression of pairwise genetic distance against pairwise geographic distance among *Dryadomyces quercivorus* isolates. Each point represents a pairwise comparison between isolates. The fitted regression showed a positive relationship between genetic and geographic distance (y = 0.2028 + 4e-5x), consistent with isolation by distance

Based on 217,240 SNP loci, window-based Tajima’s D was slightly positive, with an overall summary value of 0.4089 (Table 2; Supplemental Table 2). Boxplots were used to visualize the distribution of window-based Tajima’s D values across beetle lineages and geographic regions, with median values generally positive across population groupings (Supplemental Fig. 2). Though positive Tajima’s D values indicate an excess of intermediate-frequency variants relative to neutral expectations and may be consistent with a weak population bottleneck, balancing selection, or the effects of population structure, given the modest magnitude of the value and evidence for population structure, we interpret this result cautiously as showing little overall deviation from neutrality with a pattern broadly consistent with weak bottlenecking and/or structured genetic variation. The PHI test revealed significant evidence of recombination (*p* < 0.001) when comparing all isolates, as well as within isolates within each beetle lineage, and isolates within each region. Most AMOVA variance was assigned to the within-sample component (76.5%), with smaller components attributable to beetle lineage (9.6%) and region (13.9%) (Table 3). Multilocus genotype analysis indicated that all 32 isolates had unique genotypes (32 MLGs among 32 individuals), indicating that no clones were detected; accordingly, clone correction did not change the AMOVA variance components or interpretation. To investigate population structure and assignment of individuals, we used ADMIXTURE. ADMIXTURE cross-validation indicated that K = 1 had the lowest mean CV error, although the difference between K = 1 and K = 2 was small (Supplemental Fig. 3). At K = 2, 18 isolates were predicted to have 95% or more of their ancestry from population (1) Isolates assigned to this population were all collected from beetle lineage A. These isolates were collected from the “north”, “central”, “west coast,” and “southwest” regions of Japan (Fig. 1). Eight isolates were predicted to have 99% or more of their ancestry from population (2) All isolates in this population were collected from the “east coast” region, and their host vectors were all from beetle lineage B (Fig. 1). The remaining six isolates, at K = 2 showed mixed ancestry consistent with admixture between the two inferred populations. These isolates showed between 35 and 51% ancestry from population 1 and 48–65% from population 2. These admixed isolates originated from the “Miyake Island”, “south,” and “southwest” regions of Japan, and five of the six isolates were collected from beetle lineage A (Fig. 1). At K = 3, the admixed isolates were assigned largely to a third inferred cluster. Linear regression of pairwise genetic distance against geographic distance indicated that samples separated by greater geographic distances also tended to be more genetically differentiated (Fig. 2). The Mantel test results indicated that both geographic distance (Mantel statistic r: 0.34640 and significance: 0.001) and beetle-lineage (Mantel statistic r: 0.45900 and significance: 0.0001) are significant predictors of genetic distance, indicating that fungal population structure is associated with both spatial separation and beetle lineage. However, the Mantel test did not show that beetle lineage was spatially correlated (Mantel statistic r: 0.02426 and significance: 0.3393). The MRM analysis showed that both geographic distance (*p* = 0.0005) and beetle distance (*p* = 0.0001) each were significant predictors of genetic distance. The significant association between genetic and geographic distance indicates the presence of isolation by distance, suggesting that spatial autocorrelation may contribute to the clustering inferred by ADMIXTURE. However, because beetle lineage was also significantly associated with genetic distance and was not itself spatially structured, the observed population structure is unlikely to be explained by geography alone.

**Table 3:** Analysis of molecular variance (AMOVA) using a hierarchical analysis of isolates collected from two beetle lineages representing populations, regions representing subpopulations, and each isolate as within sample variation using the full dataset

	df	Sigma	% variance	*p*-value
Between population	1	5538.84	9.6	0.001
Within population	6	8067.43	13.9	0.001
Within samples	24	44293.19	76.5	0.037
Total	31	57899.46	100	

**Fig. 3:**
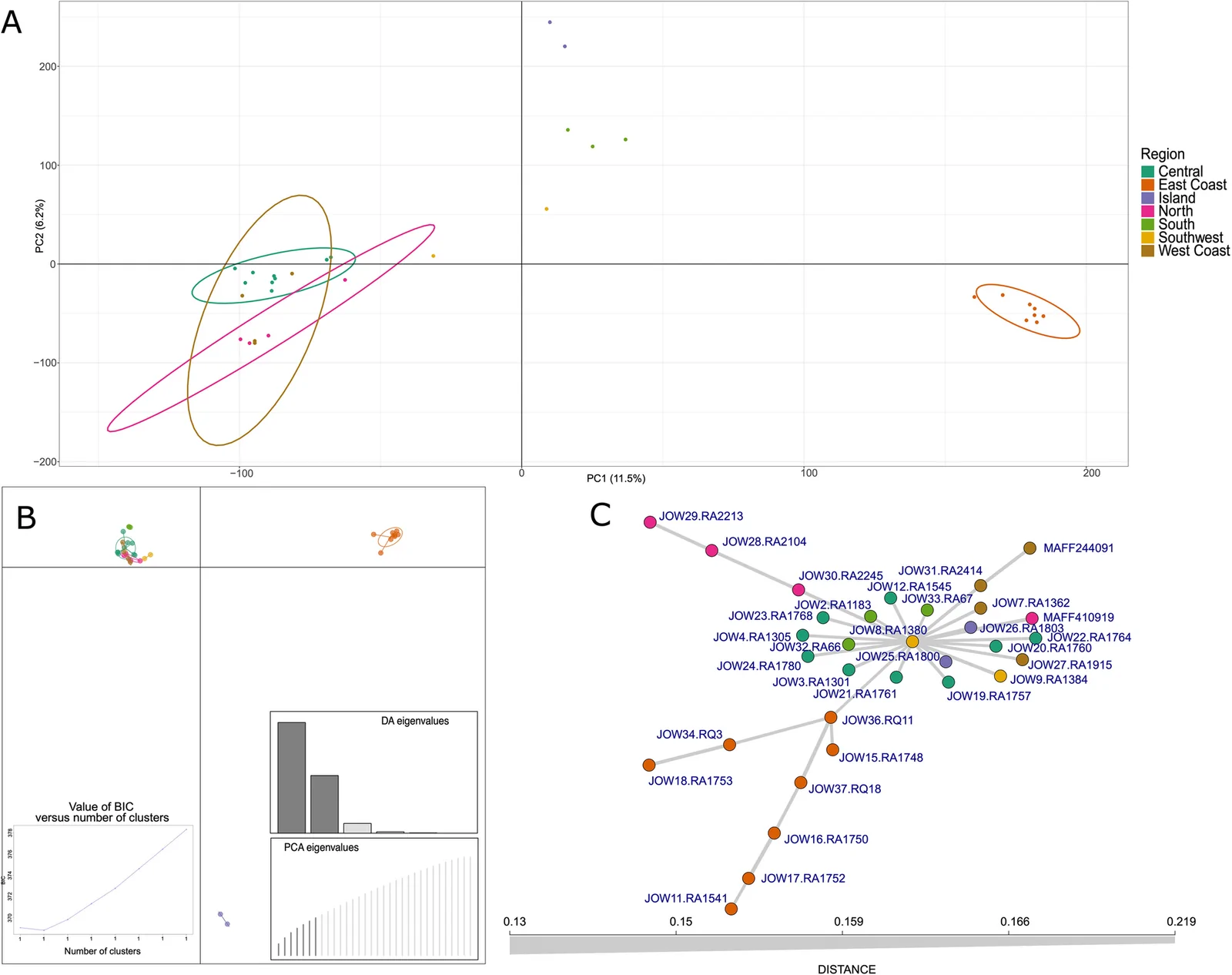
Population structure analyses show weak clustering of *Dryadomyces quercivorus* isolates by geographic region. Clustering analyses of 33 isolates are shown: (**A**) principal components analysis (PCA); (**B**) Discriminate Analysis of Principal Components (DAPC); and (**C**) minimum spanning network (MSN) based on isolates collected from two beetle lineages and colored by their geographic region of origin

**Fig. 4:**
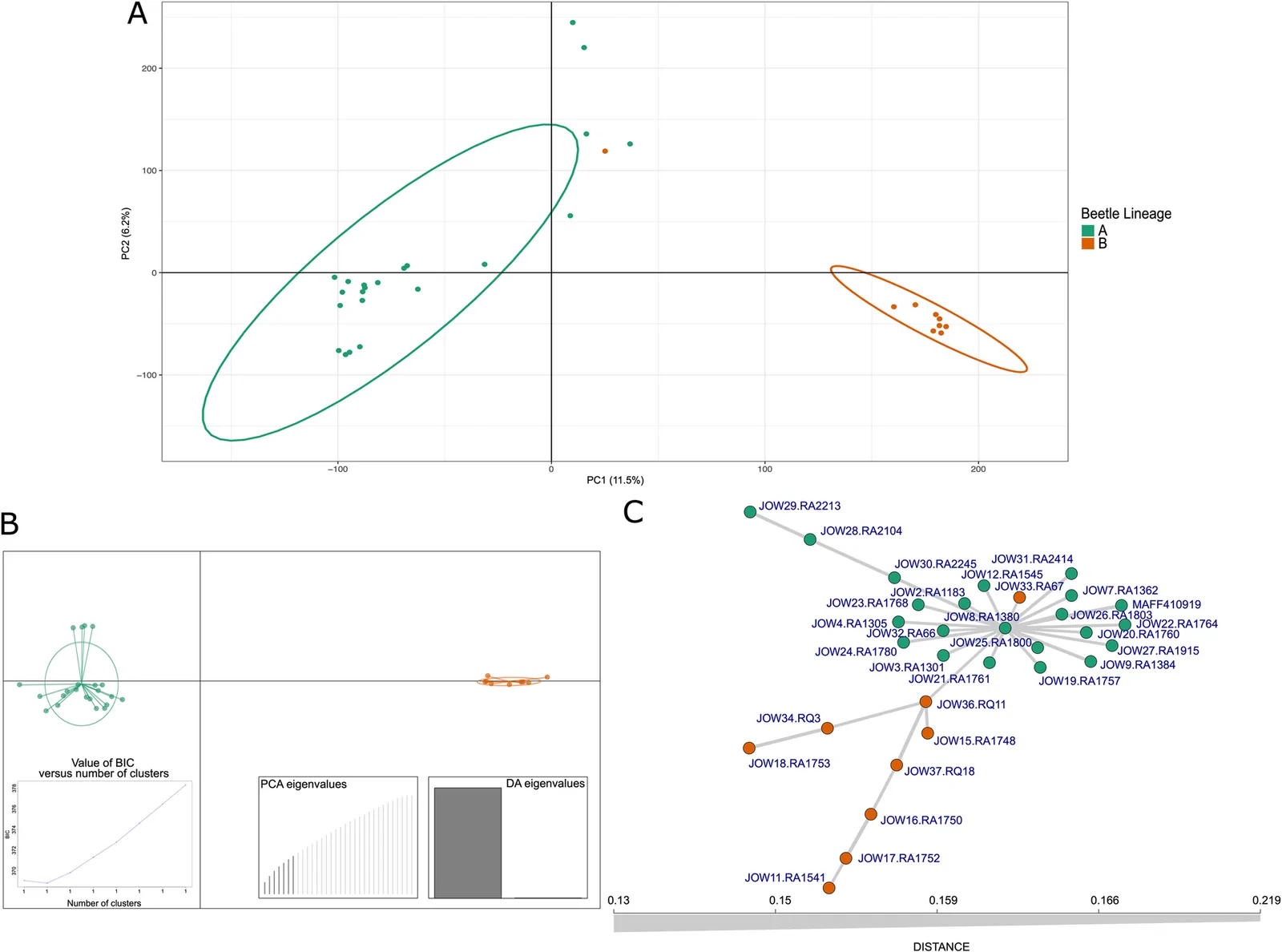
Population structure analyses show clustering of *Dryadomyces quercivorus* isolates by beetle vector lineage. Clustering analyses of 33 isolates are shown: (**A**) principal components analysis (PCA); (**B**) Discriminate Analysis of Principal Components (DAPC); and (**C**) minimum spanning network (MSN) based on isolates collected from two beetle lineages. In all panels, points represent individual isolates and are colored by their associated beetle vector lineage (lineage A or lineage B)

**Fig. 5:**
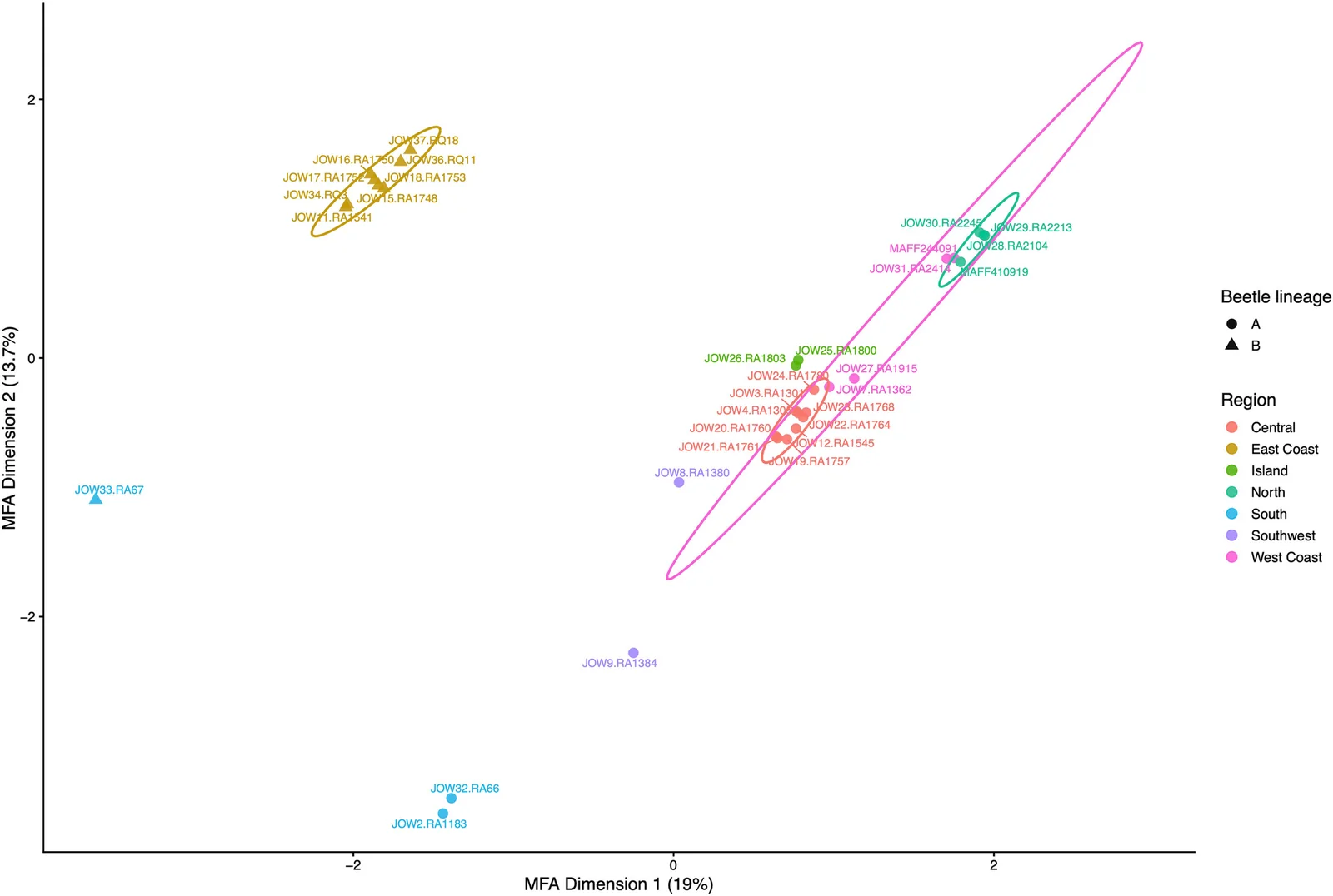
Multiple factor analysis (MFA) of *Dryadomyces quercivorus* isolates. MFA was conducted using genome-wide SNP-derived principal components together with geographic region and beetle vector lineage. Each point represents one isolate and is colored by geographic region and shaped by beetle lineage. The first two dimensions summarize the combined contribution of genomic variation and associated metadata to the observed population structure

The DAPC analysis similarly found K = 2 was the optimal number of clusters (Supplemental Fig. 4). Across analyses based on region (Fig. 3), beetle lineage (Fig. 4), or both variables combined (Fig. 5), the four clustering methods recovered broadly similar patterns of grouping. However, comparison of virulence using previously published canker length data [20] showed no clear separation between isolates that produced canker lengths significantly different from the control in earlier studies [20, 86] (Supplemental Fig. 5).

### Comparative genomics

**Fig. 6:**
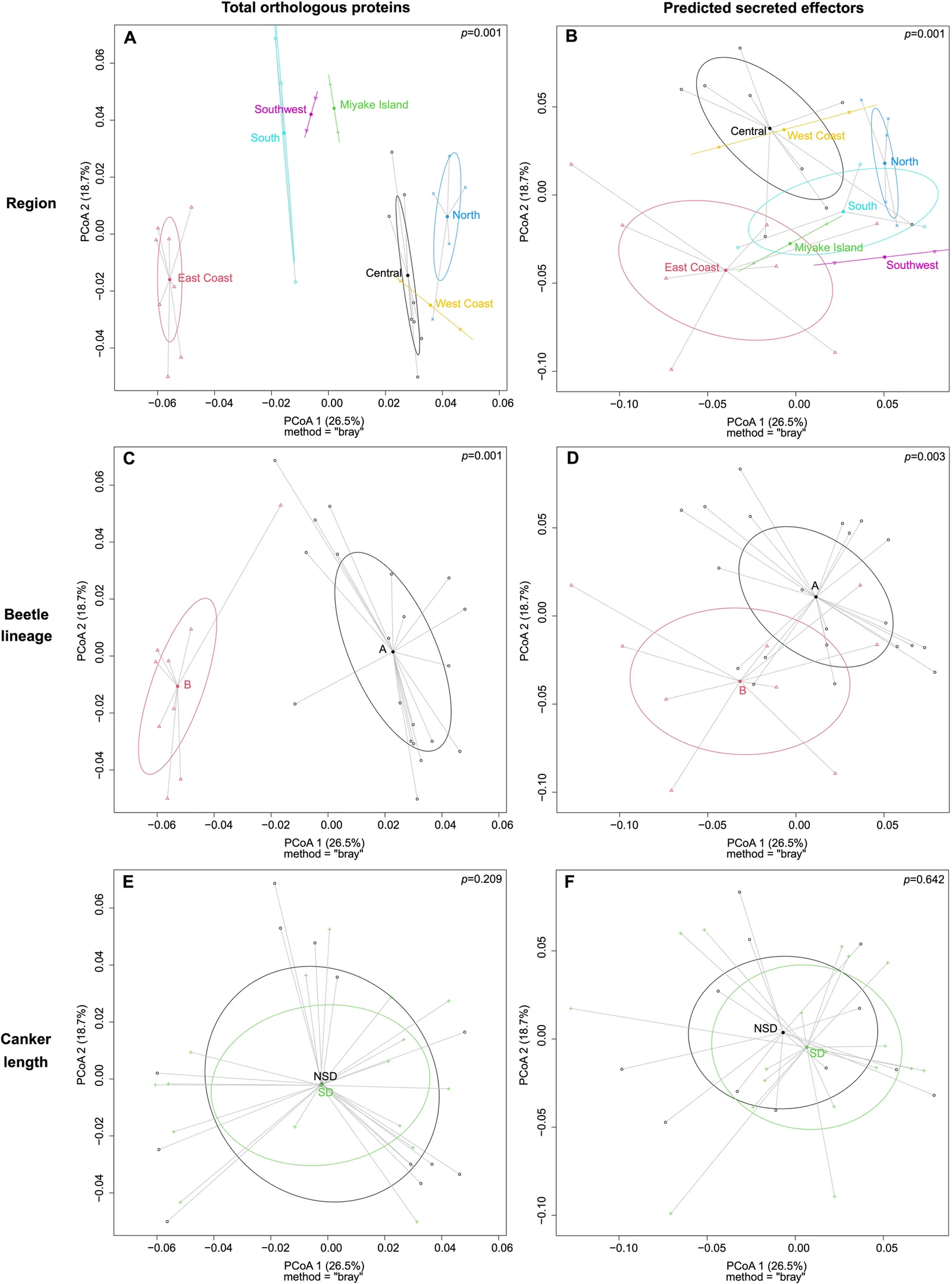
Orthogroup composition of *Dryadomyces quercivorus* differs significantly by beetle lineage but not by virulence category. Principal Coordinates Analyses (PCoA) based on Bray-Curtis dissimilarities of (left column, **A**, **C**, **E**) total orthologous proteins (**A**, **C**, and **E**) and (right column, **B**, **D**, **F**) secreted effectors of 33 *D. quercivorus* isolates. Isolates are contrasted by geographic region (**A** and **B**), beetle lineage (**C** and **D**), and whether the isolates caused significant disease (SD) on *Quercus serrata* based on canker length compared to the control (**E** and **F**). Ellipses represent 95% confidence intervals. Statistical significance between groups was assessed using PERMANOVA (p-values reported in text)

Among total proteins, OrthoFinder assigned 291,684 genes (98.5% of total) to 12,164 orthogroups. The gene content was summarized by a G50 of 33 (i.e., 50% of genes were in orthogroups with at least 33 genes) and an O50 of 4,375 (i.e., the 4,375 largest orthogroups contained 50% of all genes). There were 6,628 orthogroups with all species present and 4,125 of these consisted entirely of single-copy genes. PERMANOVA revealed that the composition of total orthologous proteins was significantly different based on region (*p* = 0.001) and beetle lineage (*p* = 0.001) but not significantly different based on virulence as measured by canker length (*p* = 0.209) (Fig. 6).

Among predicted secreted effectors, OrthoFinder assigned 2,319 genes (99.2% of total) to 93 orthogroups. Here, the G50 was 33 and the O50 was 35. On average, 71 secreted effectors were predicted in the JOW isolates. Most secreted effectors were identified as apoplastic effectors (average of 73.8%) with a smaller number of cytoplasmic (average of 13.5%) or dually-predicted effectors (average of 12.7%). The composition of secreted effectors showed a similar trend and was significantly different based on region (*p* = 0.001) and beetle lineage (*p* = 0.003) but not significantly different based on canker length (*p* = 0.642) (Fig. 6). Among the secreted effectors, an average of two PHI-base homologous genes (PHI:6417; PHI:222) and three glycoside hydrolase CAZymes GH18, GH28, and GH43 were predicted in nearly all JOW isolates, except PHI:6417 was not predicted in isolate JOW25.RA1800, PHI:222 was not predicted in isolate JOW7.RA1362, and GH18 was not predicted in isolate JOW11.RA1541 (Table 4; Supplemental Table 1). PHI-base homologous gene PHI:6417 was also predicted in the KOW pathogen genomes. Glycoside hydrolase CAZymes GH7, GH18, GH28, GH43, and GH54 were predicted in the KOW and MOB pathogen genomes (Supplemental Table 1).

**Table 4:** Predicted secreted effectors of interest for *Dryadomyces quercivorus* described as pathogenicity/virulence genes in other fungal species or carbohydrate active enzymes (CAZymes)

Gene	ID	Effector type	Description
g1572.t1	PHI:6417	Proteinaceous elicitor	Sm1 induces plant defense responses and systemic resistance
g4635.t1	PHI:222	CAZyme	PELB pectate lyase degrades pectin, specifically polygalacturonic acid
g53.t1	GH18	CAZyme	Contains chitinases of classes III and V
g6936.t1	GH28	CAZyme	Polygalacturonases that break down pectin
g6754.t1	GH43	CAZyme	α-L-arabinofuranosidases, endo-α-L-arabinanases, β-D-xylosidases, exo-oligoxylanases, or exo-α-1,3-galactanase involved in the degradation of hemicellulose and pectin

## Discussion

Our findings provide a clear genomic window into the complex interactions that define an emerging forest disease. The high-quality genomes generated in this study represent a foundational step, achieving high contiguity while matching or improving upon the completeness of currently available references. Though the genome of the JOW pathogen holotype generated in this study was 0.4 Mb smaller than the current NCBI reference genome, our assembly is more contiguous and more complete based on QUAST and BUSCO results. This size difference is likely due to a higher number of duplicated BUSCO genes in the NCBI reference, whereas our assembly resolved these into single copies. While the hybrid ONT and Illumina assemblies for the other JOW isolates were also highly contiguous, the inclusion of previously published PacBio long reads for the holotype likely contributed to the highest level of completeness. In the KOW genomes, our hybrid ONT and Illumina assemblies resulted in more contiguous genomes but did not improve on the completeness compared to the current reference. Notably, this study provides the first publicly available genome of *D. montetyi*, the primary nutritional symbiont of the Mediterranean Oak Borer (MOB, *Xyleborus monographus*), an invasive beetle recently established in the western United States [89, 90]. This genome serves as a crucial genomic baseline for monitoring the fungal population associated with the ongoing MOB invasion in North America. This baseline is essential for tracking the diversity and evolutionary trajectory of *D. montetyi* as it interacts with naïve North American ecosystems and for developing specific diagnostic tools to monitor its spread alongside its beetle vector. These high-quality genomes could be further improved by using a combination of sequencing technologies including PacBio HiFi, ONT long reads, and Dovetail Omni-C to generate chromosome-scale assemblies [91, 92]. Telomere-to-telomere references are critical for improving the accuracy of further population genomic studies, the development of diagnostic tools, and the prediction of pathogenicity/virulence genes [92, 93]. Nevertheless, the high-quality genomes presented here for both the JOW pathogen and the emerging MOB pest complex represent critical, novel genomic tools for the global research community, providing an immediate resource for conservation and management efforts.

A central finding of this study is that, although genome-wide structure was relatively weak and ADMIXTURE cross-validation favored K = 1, multiple complementary analyses revealed biologically meaningful genetic differentiation associated with beetle vector lineage, with geographic distance also contributing significantly. However, previously reported virulence does not show a clear association [20]. This highlights the deep interconnectedness of species that drives disease dynamics at an ecosystem scale. Our results show that the strongest non-random genetic differentiation detected in the pathogen broadly mirrors the two beetle lineages (Group A and Group B) recently described for the beetle vector [25], consistent with a co-structured relationship between these two organisms. These findings suggest that surveillance and quarantine efforts should be prepared to detect and manage two recently divergent vector-pathogen complexes, not a single monolithic one. While ADMIXTURE cross-validation scores suggested that K = 1 was the most probable number of clusters, the results at K = 2 revealed a biologically meaningful structure that was consistent with the DAPC analysis. The small number of admixed isolates we identified, which show ancestry from both major populations, may represent rare instances of successful long-distance dispersal and recombination between the two lineages, potentially facilitating gene flow across these structured populations. The distinctiveness of the “east coast” region, which was dominated by beetle lineage B isolates, could be consistent with a separate founder event, although broader sampling across Japan and neighboring regions would be needed to test this hypothesis. Notably, despite this clear genetic structure, we found no significant correlation between these genetic populations and the previously reported virulence levels based on canker length [20, 66]. ADMIXTURE can infer discrete clusters even when genetic variation is distributed continuously across space. In our dataset, the significant relationship between geographic and genetic distance indicates that isolation by distance is present and may partially contribute to the inferred clustering. However, beetle lineage remained significantly associated with genetic distance in both Mantel and MRM analyses, and beetle lineage itself was not spatially autocorrelated, suggesting that beetle-associated structure may also contribute independently to the observed population differentiation.

Understanding the pathogen’s genetic variation is critical for predicting its adaptive potential and its ultimate impact on forest ecosystem dynamics. Although differentiation among beetle lineages and regions was modest, the AMOVA results indicate that most variation was not partitioned among these predefined groups. This pattern is broadly consistent with previous SSR-based studies reporting limited population differentiation in JOW and KOW pathogens [32, 94]. Low genetic diversity can be consistent with a genetic bottleneck resulting from a founder effect, a pattern commonly observed in invasive species [95]. In our dataset, Tajima’s D values were generally positive, including a slightly positive overall value. This pattern may be consistent with a weak population bottleneck; however, positive Tajima’s D can also arise from balancing selection or population structure. Since genetic structure associated with beetle lineage and geography were detected in our analyses, may be consistent with weak bottlenecking and/or structured genetic variation rather than as definitive evidence of a recent bottleneck. The Korean oak wilt pathogen was described as non-native to Korea based on RAD-tag data showing low SNP diversity among genomic restriction sites [96]. While some have postulated that these are native pests and pathogens to Japan [97, 98], our observation of low genetic diversity and generally positive Tajima’s D values is also consistent with the alternative hypothesis that these pathogens experienced demographic bottlenecks, potentially associated with introduction or range expansion [99, 100]. However, this conclusion should be treated with caution due to the limited sample size in this study and the lack of isolates collected from regions outside Japan.

Despite the low overall diversity between populations, we found genomic evidence consistent with recombination, together with mating-type distributions compatible with a sexual cycle. The significant PHI test result indicates conflicting phylogenetic signal consistent with recombination. Together with the presence of both mating-type idiomorphs in approximately balanced frequencies, this suggests that recombination may occur within these beetle-associated groups. This suggests that recombination may occur within these groups; if mating occurs more frequently among isolates associated with the same beetle lineage, it could help maintain their genetic distinction. The presence of both mating-type idiomorphs in an overall ratio not significantly different from 1:1, together with PHI-test evidence of recombination, is consistent with the potential for a sexual cycle [68]. The flanking genes APN2 and SLA2 enabled identification of the MAT locus but do not themselves constitute evidence of sexual reproduction. A mechanism for this mixing is suggested by Takahashi et al. [32], who found that multiple pathogen genotypes can co-occur within a single beetle’s mycangia and gallery. From a biosecurity perspective, these findings suggest that a single intercepted beetle should not be considered a single introduction, but rather a vector carrying a reservoir of genetic diversity. This could increase the establishment risk associated with even minor quarantine breaches. Reports that ambrosia beetles are capable of acquiring additional pathogens within infected wood is a concern because it suggests that if these beetle vectors spread globally, they could acquire and transmit novel tree pathogens in new environments [101, 102].

The JOW beetle vector is not a strong long-distance flyer [14], meaning long-distance spread is likely human-mediated through the transport of firewood or untreated wood products [103]. This pathway is a classic example of the human-facilitated introductions that have spread alien species to every region of the globe [3]. The distinctiveness of the “east coast” population (Lineage B) may be consistent with a separate founder event, possibly linked to a port of entry. This model of multiple, independent introductions is a well-documented pattern in ambrosia beetle invasions; for example, population genomic analysis of the black stem borer, *Xylosandrus germanus* (Hoffmann), revealed multiple introductions into North America and Europe from different source populations within its native Asian range [104]. This underscores the need for broader genomic surveillance of both the beetle and the pathogen across Asia and the Pacific to identify their native range and track invasion pathways. The confirmation that both JOW and KOW beetle vectors have spread to mainland China [105] demonstrates that this geographic expansion is ongoing, making preemptive surveillance in other regions even more critical. As seen with laurel wilt, where broad geographic surveys identified the likely origin of *H. lauricola* [106], a similar effort is needed here.

A key goal in the genomics of forest disease is to identify the genetic determinants of virulence. While our comparative genomics analysis of putative virulence genes did not reveal a simple presence/absence pattern of genes correlating with high virulence (Supplemental Table 1), this itself is a critical genomic insight into this disease system. Unlike model pathogens such as *Fusarium oxysporum* (Schlechtendal), where virulence can be linked to discrete genomic regions like pathogenicity islands, our results suggest that virulence in *D. quercivorus* may be a more quantitative trait controlled by gene regulation or subtle sequence variation rather than the presence of large, accessory genomic blocks. For risk assessment, the absence of a clear lineage-specific genetic signature of virulence suggests that any incursion of *P. quercivorus*, regardless of its lineage, carries the potential to introduce highly virulent pathogen strains. Based on our current data, we found no evidence to support deprioritizing one lineage as clearly lower risk than the other. This suggests that the observed variation in canker length may be driven by environmental factors or subtle differences in gene expression rather than the presence or absence of specific virulence genes. However, our analysis did identify a core set of putative effectors shared among isolates, such as a proteinaceous elicitor (PHI:6417) and several glycoside hydrolases (GH18, GH28, GH43), which are homologous to genes implicated in virulence in other pathogenic fungi [107–109]. Therefore, while our genomic analysis did not identify a clear genetic basis for virulence variation, it provides a list of high-priority candidate genes for functional analysis. Future transcriptomic and proteomic studies during host infection are needed to determine if the expression levels of these genes correlate with virulence.

### Specific biosecurity relevance to the United States

The implications of these findings have direct and significant relevance for North American forest ecosystems. As established in our introduction, oaks are a keystone ecological and economic species in the U.S., forming the backbone of the hardwood timber industry and representing a ‘naïve’ host landscape with no co-evolutionary resistance to these pathogens. The history of invasive forest pathogens in the U.S., such as those causing chestnut blight and Dutch elm disease, provides a sobering precedent for the potential economic and ecological impact. This includes direct threats to biodiversity conservation, as demonstrated when the laurel wilt pathogen jumped to infect the federally threatened pondberry (*Lindera melissifolia*) [19]. The introduction of JOW could therefore pose a similar risk to the many rare and endemic *Quercus* species in North America, in addition to widespread commercial species. This risk is not abstract; the total economic costs of invasive species in North America have been quantified in the tens of billions of dollars annually [4]. Our research elevates this threat from a general concern to a specific, mechanistic risk. Our results suggest that a single beetle may introduce not just a pathogen genotype, but a genetically diverse pathogen population with evidence consistent with recombination. This could make any introduction into the U.S. exceptionally challenging to contain and could accelerate the pathogen’s adaptation to North American oak species. Therefore, our findings support heightened biosecurity and preemptive research focused on protecting U.S. timber resources.

### Management Implications

Global assessments confirm that preventing the introduction and spread of alien species is the most effective and cost-efficient solution to the invasion crisis [3]. The genomic resources generated in this study provide foundational novel genomic tools for developing a modern preventative biosecurity strategy for North America. The highly contiguous genomes provide a strong foundation for developing specific diagnostic assays for rapid detection in wood samples or trapped beetles at ports of entry. Furthermore, the SNP data generated here provides a foundation for future genetic fingerprinting efforts that could help infer the origin of any new North American incursions and support the identification of invasion pathways. The presence of both mating types underscores that prevention is the most critical strategy, as eradication of an established, sexually reproducing population would be exceptionally difficult and costly.

## Supplementary Information

Additional files 1: Supplementary Table 1 – Illumina and Oxford Nanopore Technology reads and assemblies and associated metadata of *Dryadomyces quercivorus* isolates from Japan. Annotations based on Braker predicted proteins and described secreted proteins and effectors.

Additional files 2: Supplementary Table 2 – Window-based Tajima’s D summary statistics for *Dryadomyces quercivorus *populations grouped by beetle vector lineage and geographic region. Tajima’s D was calculated in non-overlapping 10 kb windows with a 10 kb step size using biallelic SNPs. For each beetle lineage or geographic region, the table reports the mean Tajima’s D, median Tajima’s D, standard deviation of Tajima’s D, number of valid windows, and weighted mean Tajima’s D. Valid windows were defined as windows containing at least two segregating sites. Weighted mean Tajima’s D was calculated using the number of segregating sites per window as weights.

Additional files 3: Supplementary Figure 1 – Splitstree phylogeny based on single nucleotide polymorphisms of *Dryadomyces quercivorus* isolates collected from seven regions within Japan. SplitsTree was used to visualize reticulation/conflicting signal consistent with recombination.

Additional files 4: Supplementary Figure 2 – Distribution of window-based Tajima’s D values by population grouping. Boxplots show Tajima’s D estimates calculated across valid genomic windows for each beetle lineage and geographic region. Center lines indicate medians, boxes show interquartile ranges, whiskers indicate 1.5× interquartile ranges, and points represent outlier windows.

Additional files 5: Supplementary Figure 3 – Boxplot of cross-validation values from ADMIXTURE to estimate the optimal number of populations (K) ranging from 1 to 7 based on ten replicate runs. The optimal number of K was 1 based on the lowest average CV value.

Additional files 6: Supplementary Figure 4 – Discriminant analysis of principal components (DAPC) determined the optimal number of populations (K) was 2 based on lowest (BIC) value (A). The DAPC plot shows genetic structuring, with geographic origin contributing to separation along LD1 (B). The east coast isolates represented a distinct population compared to the other regions (B and C).

Additional files 7: Supplementary Figure 5 – Minimum spanning network did not show clear clustering based on significant disease as determined by significant canker length compared to mock inoculated control plants.

## Data Availability

The datasets generated and/or analyzed during the current study are available in the National Center for Biotechnology Information (NCBI) repository, in the BioProject PRJNA1235289 (https://www.ncbi.nlm.nih.gov/bioproject/PRJNA1235289). Raw sequencing reads are available through the NCBI Sequence Read Archive (SRA) under accessions SRR32677234 – SRR32677277 and SRR38229615 – SRR38229621. The datasets used and/or analyzed during the current study are available from the corresponding author on reasonable request.
